# Mucocutaneous Paraneoplastic Syndrome Secondary to Classical Hodgkin’s Lymphoma

**DOI:** 10.5811/cpcem.2019.3.41759

**Published:** 2019-04-02

**Authors:** Jenna Jurkovic, Michael Cirone

**Affiliations:** * Advocate Christ Medical Center, Department of Emergency Medicine, Oak Lawn, Illinois; † University of Illinois-Chicago, Department of Emergency Medicine, Chicago, Illinois

## Abstract

Paraneoplastic syndromes may be the only presenting manifestation of an otherwise occult malignancy. This case report highlights a patient presenting to the emergency department with an atypical, multi-system disease, ultimately leading to a diagnosis of mucocutaneous paraneoplastic syndrome secondary to classical Hodgkin’s lymphoma. Emergency physicians should maintain a high clinical suspicion for paraneoplastic syndromes when patients present with multi-system manifestations.

## CASE PRESENTATION

A 39-year-old Hispanic male with no past medical history presented to the emergency department (ED) with a chief complaint of “allergic reaction.” Pertinent review of systems included several weeks of left facial and neck swelling, pharyngitis, non-productive cough, and rhinorrhea. He was evaluated multiple times in the urgent care setting and treated with several different antibiotic regimens for bacterial pharyngitis. He later developed bilateral conjunctivitis, oral ulcers, and a solitary penile lesion. Outpatient medications were broadened to include antivirals, antifungals, and steroids. Due to persistence of symptoms, along with the development of hematuria and rectal pain, the patient sought evaluation in the ED.

**Image 1:**
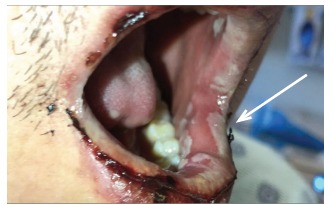
Multiple ulcerative oral lesions.

**Image 2:**
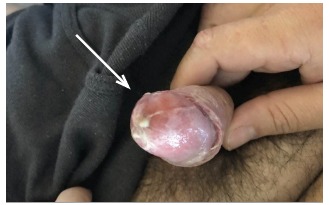
Purulent penile discharge.

**Image 3:**
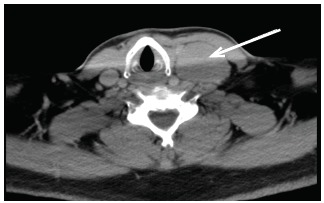
Computed tomography, axial image, demonstrating left level 2–5b neck adenopathy.

Physical examination revealed bilateral, non-purulent conjunctival injection, multiple non-painful ulcerative oral lesions (Image 1), tender left cervical lymphadenopathy with edema, and a single non-ulcerative penile lesion with purulent discharge at the glans (Image 2). Vitals were within normal limits. Laboratory studies revealed a slight leukocytosis, but the remainder of labs, including inflammatory markers, were unremarkable. Computed tomography of the neck demonstrated left-sided cervical adenopathy suspicious for neoplasm (Image 3).

## DISCUSSION

The multisystem involvement of the patient’s clinical presentation yielded a broad differential diagnosis including allergic, autoimmune, infectious, or neoplastic etiology. Inpatient workup including lymph node, fine-needle aspirate and a biopsy of the lip established the diagnosis of a mucocutaneous paraneoplastic syndrome secondary to classical Hodgkin’s lymphoma with mixed cellularity and Epstein-Barr virus positivity.

It is estimated that 8% of cancer patients are affected by paraneoplastic syndromes.^1^ These syndromes often present with multisystem manifestations secondary to humoral substances produced by the tumor. In some cases, the paraneoplastic syndrome may be the only physical manifestation of an otherwise occult malignancy.^2^ Emergency physicians should maintain a high clinical suspicion for paraneoplastic syndromes when patients present with atypical multi-system endocrine, infectious, dermatologic, or rheumatologic disease. Timely detection, diagnosis and treatment of an otherwise occult malignancy, can significantly improve survival.^2^

**CPC-EM Capsule**

What do we already know about this clinical entity?

*Paraneoplastic syndromes may be the only presenting manifestation of an otherwise occult malignancy*.

What is the major impact of the image(s)?

*Multisystem abnormalities should raise suspicion for paraneoplastic syndrome*.

How might this improve emergency medicine practice?

*Emergency physicians should maintain a high clinical suspicion for paraneoplastic syndromes when patients present with atypical, multi-system manifestations, and this should warrant further diagnostic studies*.
